# Trainee doctors’ perceptions of the surgeon stereotype and its impact on professional identification: a qualitative study

**DOI:** 10.1186/s12909-022-03765-1

**Published:** 2022-10-04

**Authors:** David Wainwright, Michael Harris, Elaine Wainwright

**Affiliations:** 1 Department for Health, University of Bath, Bath, UK; 2 Institute of Primary Health Care Bern (BIHAM), University of Bern, Bern, Switzerland; 3 Epidemiology Group, University of Aberdeen, Aberdeen, UK

**Keywords:** Professional identity, Careers in surgery, Gender

## Abstract

**Background:**

The demography of the medical profession is changing as more women join the workforce. Traditional assumptions about the personal qualities required to be a successful surgeon may change as more women join the specialty. While exploring the attitudes and beliefs of doctors in their second ‘Foundation’ year of post-graduate training (FY2) about their choice of specialty, evidence emerged about how the stereotype of the surgeon influences professional identification and beliefs about person-specialty fit.

**Methods:**

Qualitative telephone interviews with 24 FY2 doctors, 17 women and 7 men, in South-West England.

**Results:**

Many participants reported exposure to stereotypes about the personal qualities desirable in a surgeon. Senior doctors and other trainees were the primary source of these stereotypical views. Experience on surgical placements could either reinforce stereotypes or challenge them, the latter particularly where senior surgeons provided positive role models. As more women enter the surgical specialties, they are increasingly challenging the traditional stereotype and sub-culture.

**Conclusion:**

Gendered stereotypes about surgical roles persist, and for some this can hinder professional identification with the role. Positive role models and mentoring can encourage and support women who are interested a surgical career to identify with the role, but there is a need for a broader debate encompassing job redesign and surgical identities.

**Supplementary Information:**

The online version contains supplementary material available at 10.1186/s12909-022-03765-1.

## Introduction

The year 2021 marked the 30th anniversary of the Royal College of Surgeons of England’s ‘Women in Surgery’ (WinS) initiative, which aims to promote and support women surgeons. The WinS website includes statistics on the gender mix in surgery [1]: in the United Kingdom (UK) in 1991, 3% of consultant surgeons were women, rising to 13% by 2020. A 2017 systematic review and meta-analysis showed that women were proportionately less likely to apply for and complete surgical training [2]. While progress towards gender equality, in terms of the number of applicants for surgical training, and the number pursuing and succeeding in surgical careers, has been made, there is a considerable way yet to go [3, 4].

The empirical evidence points to several structural and cultural factors that influence women’s choice of a career in surgery, the advancement of those who become surgeons, and their likelihood of remaining in the surgical specialties. Structural factors include long-hours [3], shift-work, limited opportunities for flexible working [5, 6], and a highly competitive pathway to advancement, which may disadvantage those who take career breaks [7]. These structural characteristics may discourage men as well as women, but it has been argued that women are particularly disadvantaged because they are more likely to take on childcare and other caring responsibilities, which may lead to career breaks and career choices that are easier to reconcile with these responsibilities [3, 8]. This has led some commentators to call for improvements in childcare, job-sharing, and flexible contracts [4, 9].

Other studies have found that cultural factors may be a more significant deterrent than structural ones. A survey from the United States of America found that women were less likely to be deterred from surgical careers by structural factors than by perceptions of surgery as an ‘old boys’ club’ [10].

Beliefs about the position of women in society, and assumptions about the characteristics that women are likely to have, may lead to discrimination against women in surgical training, recruitment and advancement;these may also be internalised by women themselves as part of their self-identity, leading to career choices outside the surgical specialties [6, 11]. A survey of surgical trainees found that bullying and undermining behaviour were common in the surgical specialties, with sexism being the most common manifestation, reported by 66% of women informants [12].

Empirical studies have identified the structural and cultural factors that women perceive to be obstacles to a career in surgery but how can these factors be theorised? Elsewhere, we have developed a theory of ‘person-specialty fit’ to explain the process by which trainees reflect on the characteristics of a particular specialty and decide whether they have an appropriate ensemble of personality traits, personal attributes and lifestyle preferences, to succeed and be satisfied with a career in that specialty [13, 14]. An important aspect of person-specialty fit is the process of professional identity, which others have described as ‘a complex structure that the individual uses to link their motivations and competencies to their career role’ [15:370], and as ‘ways of being and relating in professional contexts’ [16]. At its simplest, this process might be conceptualised as a simple matching of personal qualities and preferences with the characteristics and demands of a given specialty, but Goldie [16] proposes a far more complex and socially negotiated process in which doctors develop an understanding of themselves and of different specialties through interaction with other healthcare professionals, patients, and wider cultural representations, including medical television dramas [15]. While theories of person-specialty fit and professional identity recognise that these shared assumptions about how doctors should be are socially constructed narratives or stories, they tell us little about the extent to which they are grounded in functional necessity, for example, the competencies and personal qualities required to perform the role successfully, or, whether they are simply cultural stereotypes that are peripheral to role performance.

Stereotyping refers to the set of expectations that people might have about individuals who belong to a particular group. These expectations include characteristics, attributes, competencies and behaviour. Stereotypes can be accurate, and useful where quick decision-making is required, but stereotypical thinking can result in a tendency to over-generalise and assume that all members of a group must share the same characteristics and patterns of behaviour, even in the face of contradictory evidence. It is this tendency to over-generalise that implicates stereotypes in prejudicial and discriminatory decision-making [17, 18], but stereotyping may also have consequences for professional identification, for example, leading some doctors to turn away from a career in surgery because they do not identify with the prevailing stereotype of the surgeon [11, 19, 20].

While stereotypes about surgeons may have played a role in reducing women’s entry to surgical specialties and their subsequent advancement, this tendency may be partly countered by the example set by successful women surgeons who act as role models [21]. A qualitative study of recently recruited academic surgeons in Canada found that the changing gender balance and a generational shift in beliefs were changing the culture of academic surgery [8]. Both men and women participants reported that departmental expectations regarding their job performance were not influenced by gender. Women struggled more than men in achieving a satisfactory work-life balance, but there were generational differences in attitudes to work-life balance, with both men and women participants valuing it more than their senior colleagues [8].

The literature suggests that surgery is a specialty in flux, still essentially male-dominated, but changing as more women become surgeons and rise to senior positions. Given these changes, it is important to ask what impact they might be having on trainee doctors’ perceptions of the specialty and their professional identification with it. What are their views of the current stereotype of the surgeon? Do they see these stereotypes as gendered? Do stereotypes affect their professional identification with surgery, and if so, how are these processes negotiated and contested? Using data from an earlier study of specialty choice among trainee doctors [13, 14], this article addresses the above issues, focusing on the following research question.

### Research question

To what extent and by what processes do trainee doctors’ perceptions of the stereotype of the surgeon influence their professional identification with surgical specialties?

## Methods

### Design

The qualitative methodology adopted in this study followed Braun and Clarke’s Thematic Analysis [22–24], using semi-structured interviews to explore participants’ views and experiences.

### Participants and recruitment

The sampling frame (n = 262) was composed of doctors who had completed their medical under-graduate degree, and were in their second year of post-graduate training, the second ‘Foundation’ year (FY2), in the South West region of the UK. Administrators, employed by the organisation responsible for post-graduate medical education in the region, emailed all 262 FY2 trainees in the region, with a study information pack and the research team’s contact details. Interested doctors then contacted the research team for any further information, before giving informed consent. Everyone who contacted the research team went on to participate (n = 24). Purposive sampling does not aim to be demographically representative of the sampling frame, but it is worth noting that we recruited 17 women participants and 7 men, when the sampling frame comprised 54% women. This limitation is considered in the discussion section. A gift voucher was offered in token of thanks for participation. We interviewed and conducted the analysis sequentially, so that early findings could inform later interviews by modifying the schedule of questions [25].

### Compliance with sex and gender equity in Research (SAGER) guidelines

We have read the SAGER guidelines [26] and complied with them. The words man and woman are central to our study and are used throughout this article. During the telephone interviews we asked participants to self-identify their gender orientation. All identified as either men or women, and these are the terms we have used to refer to the participants and to the broader social groups that share this identity. Our concern is with gender as a set of socially constructed roles, behaviours and identities, rather than with biological sex associated with physical and physiological attributes. Non-binary gender identities may be relevant to the surgeon stereotype and its influence on professional identification, but as none of our participants identified as non-binary, or referred to these identities when interviewed, we have not referred to them in this article.

### Data collection

The semi-structured interview questions were designed following a review of the existing literature on what influences trainee doctors’ career choices, and discussion among the research team (see supplementary information file for interview schedule). The interview schedule was piloted with two FY2 volunteer doctors, who suggested no substantial changes. The semi-structured interview format meant we could ask participants the same questions whilst also probing further if participants provided leads. DW and EW conducted interviews over the phone at a time of the participant’s choice; calls lasted on average 40 min. All interviews were digitally recorded and transcribed verbatim. DW and EW then anonymised transcripts before undertaking analysis.

### Data analysis

We (DW, EW and MH) used Thematic Analysis to structure data collection and analysis [22–24], using an inductive approach so that themes were generated from the data rather than from a pre-conceived coding scheme. We immersed ourselves in the data through repeated transcript readings and discussed our first interpretative notes together, (DW, EW & MH). We coded all data across the dataset, and all transcripts were coded by at least two research team members. We compared our interpretations, and jointly refined themes and sub-themes, debating differences until we reached agreement and had defined, named and exemplified all the themes. Quotations have been chosen to illustrate key themes, and the number attached to each quotation is a unique participant code.

### Positionality statement

Holmes [27] suggests that all qualitative researchers should overtly declare their position in relation to the topic of their research and those who participate in it. Positionality is a complex concept which includes: relevant demographic characteristics such as gender, class and ethnicity; ontological and epistemological standpoint; and pre-existing assumptions and beliefs about the phenomenon being studied. The aim in declaring positionality is not to eradicate bias, (which can never be fully achieved), but to reduce bias and enable others to assess the extent to which the researcher’s position may have influenced the interpretation of data. Central to this process is the extent to which the researcher adopts an ‘insider’ or ‘outsider’ perspective, that is, the extent to which the researcher is a member of the group being studied.

Our research team comprises two men and one woman, all of whom are white British and aged 50 or above. All of us are University based researchers. One of us is also a semi-retired General Practitioner. One of us combines a full-time career with motherhood. Our ontological and epistemological standpoint in relation to this project can be described as critical realist, in that we believe stereotypes and professional identities are socially constructed through interaction, but that they are embedded in institutionalised structures and practices. We have reflected on our pre-existing assumptions about what are the desirable qualities for a surgeon to have, the right of women to pursue a career in surgery without discrimination or prejudice, and the extent to which institutionalised obstacles to their participation can and should be removed. We are not immune to the prevailing cultural stereotype of the surgeon but have critically engaged with its adequacy and validity and have attempted to set these value judgements aside when analysing the data. Our position in relation to the participants is largely that of the outsider – none of us are trainee doctors considering a career in surgery – but not exclusively so, as one of us is medically qualified, and one of us has experience of combining a career with motherhood. There are differences as well as similarities in our respective positions and we have used the former to generate discussion in our choice of questions and interpretation of the data. Selecting what to report is an inescapably partial process but where possible we have included verbatim quotations to enable participants to speak for themselves rather than through our mediation.

## Results

Of the 17 women who participated, 5 intended to specialise in surgery, compared with 1 of the 7 participants who were men. A further 5 participants were undecided about their choice of specialty. Table 1 describes the gender and choice of specialty for each participant.

**Table 1:** Participant gender and specialty choice

Participant codes	Gender^a^	Intended specialty
FD1	M	Psychiatry
FD2, FD4, FD10, FD12, FD15,	W	Surgery
FD3, FD8,	W	General Practice
FD5, FD16, FD20	W	Core Medical Training
FD6	W	Psychiatry
FD7	W	Anaesthetics
FD9	W	General Practice or Psychiatry
FD11, FD24	M	Anaesthetics
FD13	M	Critical care
FD14	M	Surgery
FD17, FD18, FD19, FD22	W	Undecided
FD21	M	Core Medical Training
FD23	M	Undecided

^a^W = Woman, M = Man

The key themes identified were: surgeon stereotypes; gendered stereotypes; perpetuation of stereotypes; placements; role models; and challenging the narrative. Table 2 illustrates the codes included in each theme.

**Table 2:** Codes and themes

Codes	Themes
Banter about surgeons; the typical surgeon; characteristics of surgeons; cultural representations of surgeons	Surgeon stereotypes
Male attributes (physical); male attributes (psychological); female attributes (physical); female attributes (psychological); gender and surgery	Gendered stereotypes
Implicit gender assumptions; experiences on placements; gendered exclusionary practises (structural); gendered exclusionary practises (cultural).	Perpetuation of stereotypes
Experiences on placements; differences between placements; ‘good’ and ‘bad’ placements.	Placements
Female surgeons as role models; male surgeons as role models; female surgeons adopting ‘male’ attributes.	Role models
Choosing surgery; more female surgeons will change the stereotype; surgery as a challenge for women; changing attitudes of younger doctors	Challenging the narrative

### Surgeon stereotypes

Many participants perceived that different specialties had different stereotypes:

I do think that each job comes with their - its stereotype of a person who would fit well in that specialty […] they would say yes, I’ve been thinking of orthopaedic surgery because I think I’m this kind of person. (FD14)

The stereotype of the surgeon was not always perceived to be off-putting or negative but rather a description of attributes that might be appropriate for the role:

So I think there’s certainly a bit of a stereotype about surgeons that they are the more confident, extroverts, out-spoken characters. And you have to make quite big decisions very quickly, things like that. (FD18)

There was, however, a degree of ambivalence regarding the surgeon stereotype, with some feeling that these characteristics could shade into arrogance and egotism:

I think the surgical stereotypes can be quite negative in terms of surgeons losing a lot of their medical knowledge, but when they train perhaps being kind of oafy, sometimes arrogant. (FD20)

Some of these stereotypical attitudes were seen as being necessary for the job:

I think if you’re not a bit pushy and self-confident then you won’t be good at surgery work. (FD4)

While some referred to surgeons losing their medical skills and having a limited capacity to engage with patients, not all saw this stereotype as problematic, and it was often discussed in humorous terms:

[The surgeons] sort of think, “We can fix everything from our patients with just cutting.” […] On the medical side […] the stereotype is more, “Oh, the surgeons are all mindless people, they just like cutting, they don’t know any medicine.” (FD3)

Assertiveness and competitiveness were seen, by many, as essential traits for a surgeon:

I think you do have to be assertive, obviously. I think you have to fight your way into theatre. You are really in competition with each other at that [trainee] level. (FD20)

### Gendered stereotypes

While the above attributes can be found in women and men, we found an assumption that the stereotypical surgeon was likely to be male, and that women not only lack the necessary attributes, but also would not want to become surgeons:

I think a lot of their idea of being a surgeon is this big guy who has done all this stuff whereas I am quite a short girl. And I think lots of them looked at me and didn’t think I could be a surgeon. And then some of the consultants would just assume, they would say to me and my colleagues, “Oh, you’re girls so you don’t want to do surgery.” (FD10).

In a male-dominated specialty, the established ways in which colleagues relate to each other, their manner, language and etiquette, may be different to that found in some other specialties. One participant described this as a macho environment, which she found off-putting:

… there’s a bit of a stereotype of surgeons being brash, and the whole environment being quite macho. To a certain extent, I’ve seen that to be true, and it would put me off surgery if I was actually interested in it. (FD20)

There may also be factors that do not fit with the needs of women, for example, in relation to childcare. In the following quotation the participant refers to the experiences of a fellow trainee:

And she was having a child while she was completing her core surgical training and people sounded just so unsupportive about her going part-time and about her abilities as a surgeon. … And really if she wanted to be a trainee she needed to knuckle down and there was no acknowledgment of the fact that she had a new baby and she was breast-feeding and expressing at work and she was really bruised by it. (FD6, Female)

However, the feeling that ‘your face has to fit for surgery’ could be a problem for men as well as for women:

And then they would encourage particularly the boys to go into surgery but again, boys they thought looked like surgeons. So if they had a smaller guy….. umm, they wouldn’t necessarily….it was quite odd. (FD10)

As one participant observed, the male culture of surgery may date back to its origins in the barber shops:

I guess if you go all the way back then it was barbers being surgeons and barbers are guys. … And actually, most of my friends who are girls do not want to do surgery. …Whereas guys I know are more inclined to do more logical or quick fix specialties. (FD10)

### Perpetuation of stereotypes

Participants reported a common assumption that women could not become surgeons, or would not choose to do so:

… a lot of the time when I would say, “I want to do surgery,” and people are usually really light-hearted, but they would still say something like, “You know you are a woman right?” or, “there aren’t any women surgeons here” or, “it will be much more difficult for you because you are a woman”. (FD12)

Such overt expressions of sexism were rare. More common was a feeling of just not fitting in with the dominant surgical culture, or not matching up to the expected personality type. While some male participants also reported this, the gendered character of the surgeon stereotype made it harder for women to match up to these assumptions:

I think the kind of atmosphere around it didn’t fit my personality necessarily. I wasn’t assertive enough to be a surgeon, I think. (FD20)

It is not just men who perpetuate the belief that surgery might not be a wise career choice for a woman. Even when offering support and encouragement, female trainee surgeons could still reproduce the belief that a career in surgery may not fit with traditionally female childcare duties:

… there is a lot of support from some of the core trainees who are girls and who I spoke to. And I spoke to them about being a girl and wanting to do surgery and the way you are treated. And they were very keen for me to do it but they said, and I have received comments like this, about, “Are you worried about having children in the future, or are you worried about taking time out in the future or if you want to have a life?” (FD10)

### Placements and role models

While placement experiences could reinforce traditional gendered stereotypes of surgeons, they could also provide experiences which challenged them, not least by providing women trainees with the opportunity to test themselves and discover their own capabilities:

And it was only in my final placement where most of the other competitive cut-throat type of surgeon characters were not around the theatres as they were busy revising and I was still going into the theatres that I actually got the chance to get involved in surgery… And that has grown more and more because I have got more exposure basically because I think you don’t get all that much exposure to surgery as a medical student especially if you don’t stick out. (FD4)

A key factor influencing whether a surgical placement reinforces or challenges gendered stereotypes is the presence, or absence, of women surgeons. Here, a female trainee who had described negative experiences at a male-dominated surgical placement describes how a different placement in which female surgeons were present offered an alternative perspective:

I don’t get that in [CITY] at all as there are so many women in surgery here. (FD12)

The presence of women surgeons challenges the gendered stereotype and opens up the possibility of a career in surgery. Women surgeons could also provide support and encouragement to trainees who were considering a career in surgery:

One of the core trainees, she was on an upper GI specialty, but I got friendly with her and she was like “Yes, this is great, you’re gonna do surgery! Come and assist me with this appendix...” and really kind of put me under her wing a little bit. I think that was one of the main turning points when I was like “Oh gosh, yeah, maybe I could be good at this, maybe”. (FD15)

Though successful women surgeons can provide strong role models for trainees considering a career in surgery, it would be wrong to assume that men cannot provide positive advice and support:

I remember speaking to my educational supervisor who’s a man and saying “Sometimes I worry is surgery still a bit of an old boys club?” I knew you had a slight disadvantage if you’re female. And he just rubbished it completely and was like “That’s actually nonsense”. (FD15)

The same participant also demonstrated that the example set by women surgeons is not always unequivocally positive:

One of my supervisors was actually a female general surgeon and she’s kind of doing it all … She’s kind of in my mind she’s quite a strong… she’s quite terrifying to be honest. If she likes you, she’ll be fine but if she doesn’t like you, you’re in trouble. I would like to be as successful as her, but hopefully maybe be a bit nicer? I’ll try anyway. (FD15)

Some trainees’ preconceived negative ideas about surgery and surgeons changed when they had surgical placements:

Well, I have had some more horror stories from other hospitals. I think perhaps I have been lucky in that I really enjoyed working with the team that I was in during surgery last year and I had very supportive consultants. (FD2)

### Challenging the narrative

While our findings suggest that traditional gendered stereotypes regarding surgeons continue to influence trainee doctors, not all female trainees were put off, and some could identify with surgeons and enjoy the prevailing culture:

I love the camaraderie that comes, I think more with surgery, than with medicine. I like the banter, I like the jokes and things like that. I think I get on probably better with surgeons. (FD15)

The need to overcome the challenges of a surgical career could be attractive:

For me, one of the reasons that I wanted to do surgery is I think it will be a challenge for me because I wouldn’t say I was a natural born surgeon. … I want to do something that challenges me for my career, as well, so that’s another reason of why I wanted to do it. (FD15)

Some women had the self-confidence to relish the challenge of overcoming the stereotypes:

Why can some people do it then why not me? And then obviously because I am competitive if you are going to tell me I can’t do it then I am going to do it. (FD12)

The women participants in the study that planned to become surgeons were aware of the challenges they faced, but also recognised the potential for the specialty, and for traditional gendered stereotypes, to be transformed. The high proportion of women students entering medical school was seen as a powerful driver of change:

Surgeons have to adapt to cope with the what’s now a 70% female medical school intake. […] So I think it’s easier than it ever has been for women to go into surgery. But it still doesn’t mean it’s the easiest specialty for that life-work balance. (FD2)

There was recognition that the process of change was already happening, not least because of the example set by the many successful women in surgery:

… the further back you go, the more male dominated it was. … I don’t think that’s true now at all. There are a lot of fantastic female surgeons at [CITY]. I do wonder if that also played a part, that it’s just not a traditional career path. (FD20)

This increase in self-confidence has not only led more women trainees to believe that they can effectively perform the role of surgeon as it stands, but also that they will be able to transform the profession from within, in ways which challenge the traditional gendered stereotype of the surgeon:

I wrestled a little bit with the fact that sometimes surgeons are seen as a bit, I don’t know, as the jocks of medicine. They’re kind of like “Oh I won’t listen to somebody’s chaffs. …”. I absolutely don’t want to be like that. … I wrestled with that a little bit but I’m hoping I’m gonna be the type of surgeon who’s still able to listen to somebody’s chaffs. (FD15)

## Discussion

### Interpretation of the results

Our research question asks to what extent and by what processes do trainee doctors’ perceptions of the stereotype of the surgeon influence their professional identification with surgical specialties? The findings suggest that despite the increasing number of women entering the profession, the traditional stereotype of the surgeon persists, and that while this is problematic for some male trainees, the gendered nature of the stereotype poses particular difficulties for women in terms of their professional identification with the role.

Central to this difficulty is that many women doctors look at the stereotypical characteristics ascribed to the role of surgeon and think ‘that’s not me.’ Whether this discourages them from entering the specialty lies beyond the scope of this small qualitative study, but other studies suggest that it may, for example, Gargiulo et al. [10] found that women were discouraged by perceptions of the ‘surgical personality’ and the belief that surgery is ‘an old boys’ club.’

The theories of person-specialty fit [13, 14] and of professional identity [15, 16] remind us that although such stereotypes are socially constructed narratives, they are also grounded in structural factors and hierarchies of power. It is not simply that the stereotypical surgeon is presented in a way that some women find difficult to identify with, but also that the ways in which surgery is organised and institutionalised reinforce and support the stereotype, for example, lack of part-time or job share opportunities, shift-work, and difficulty in taking career breaks [8] not only act as barriers to women who want to combine their career with childbirth and childcare, they also reinforce the belief that surgeons are not the sort of people who desire such a balance. Our findings reveal how concepts such as ‘male dominated’ or ‘macho’ culture refer not simply to prevailing attitudes, beliefs and behaviours, but to structural factors that disadvantage women who want to combine their career with motherhood.

Hill et al. [19] have explored how these stereotypes become internalised, suggesting that women surgeons experience a conflict between the prevailing identity of surgeon and that of woman/mother, noting that women in surgery are often expected to show ‘masculine traits’. They suggest the need to develop new identities and narratives that enable women to reconcile the demands of career and family. Our findings demonstrate the ways in which women surgeons are beginning to challenge the narrative around the traditional gendered stereotype, finding new ways of combining their multiple identities, but it is important to recognise that this challenge takes place within, and is constrained by, a set of institutionalised structural barriers that cannot be overcome simply be thinking about them differently.

Many studies and commentators have highlighted the importance of role models and mentors in supporting women in surgery [3, 20, 21, 28, 29]. Role models influence by setting an example, while mentors provide direct guidance, advice and support. Our findings broadly support the view that encountering senior surgeons who are women can be positive because it demonstrates that women can and do have successful careers in the specialty, but also suggest that the function of role models is more nuanced. Women who succeed in surgery by subscribing to the traditional stereotype may alienate those who do not identify with it. Conversely, senior male surgeons who challenge the traditional stereotype may play a role in encouraging women into surgery. Thus, surgical placements can be positive or negative for women trainees, depending on the composition of the surgical team and the prevailing sub-culture. It is not just the availability of mentors and role models, but the nature of the example they set, and the guidance they provide, that is central to supporting women in surgery.

Our findings also suggest that not all women are put off surgery by the traditional stereotype and male dominated sub-culture: some see it as a challenge and have the self-confidence to face it. There is also a growing awareness that the number of women in surgery is rising and that this may bring about changes in the surgical sub-culture and stereotype. Some women trainees are actively challenging the narrative about how surgeons should think and behave. This transformative capability that some young women believe they can bring to surgery raises the possibility that, as the number of women surgeons increases, not only will traditional stereotypes be increasingly challenged, but the ways in which the specialty is practised may also be changed.

Women are not simply pushed out of surgery by a set of unfavourable structural constraints and sexist attitudes, but actively engage in a process of appraisal and reflection that may entail challenging assumptions and entrenched expectations, rather than passively receiving them. Surgical placements are the domain in which professional identification and person-specialty fit are negotiated and contested. The outcome of this negotiation is open-ended, but our findings suggest that the availability of positive role models can provide encouragement, support and guidance for women who want to become surgeons.

Our findings raise the question as to whether structural constraints, relating to how surgery and surgical training are organised and practised, are objective and largely immutable characteristics of the job, or merely conventions that emerged at a time when nearly all surgeons were men. Further research is required to establish whether these aspects might be open to job re-design.

Initiatives to encourage and support women may well have helped to increase the rate of change, but our findings raise more fundamental questions about the gendered nature of surgeon identities and job design. How many of the attributes and behaviours ascribed to the traditional surgeon stereotype are positive and beneficial, and how many are simply an outdated legacy of the past? There is a need to map surgeons’ attributes onto health outcomes and clinician wellbeing. There is also a need for a broader debate, not just about the extent to which jobs in surgery can be re-designed to suit the needs of women [28], but also how the increasing number of women in surgery can and should lead to changes in the surgeon’s identity [7].

### Limitations of the study

We used a small purposive sample which limits generalisability of the statistical probabilistic kind, although the rich findings and carefully described population may aid transferability [30]. This study was based in one region, and interviews with Foundation doctors working in other areas may have identified additional themes. However, the doctors had graduated from a variety of medical schools in different parts of the UK. Our sample comprised 17 women and 7 men which is not representative of the gender split in our sampling frame, 54% of whom were women [31]. Even so, the inclusion of 7 trainees who were men is not insignificant. The prevailing stereotype of the surgeon was reported as problematic by some of the men participants, but it was not nearly as salient for this group as it was for the women participants, thus only one of the quotations we have included is from a male participant. The quotations we have included were chosen because they illustrate the themes well, and we believe that it would be misleading to either increase the number of quotations from men participants simply because they are men, or to imply that the study only explores the woman’s perspective.

## Conclusion

This study explored the extent to which trainee doctors’ perceptions of the stereotype of the surgeon influence their professional identification with surgical specialties. Our findings suggest that the stereotype of the surgeon persists, and that while this can negatively influence professional identification among trainees who are men, the phenomenon is far more salient for women trainees. While some of the women in our study were put off surgical specialties by structural constraints, cultural factors and specifically stereotypes were the dominant themes that emerged. The number of women in surgical careers continues to increase but the pace of change may be hampered by the persistence of gendered stereotypes among junior doctors themselves, as well as in some of their more senior surgical colleagues. Positive role models and mentoring can encourage and support women who are interested in a surgical career to identify with the role, but there is a need for a broader debate encompassing job redesign and surgical identities.

## Electronic supplementary material

Below is the link to the electronic supplementary material.

Supplementary Material 1

## Data Availability

We are concerned that were we to store the anonymised data in a public access depository, there is material which would enable individuals to be identified by their family, friends and colleagues. We are willing to make the anonymised transcripts available to researchers from legitimate research establishments on condition that they are not published in their entirety. Please direct requests to the corresponding author.
